# An Automated Online Measure for Misophonia: The *Sussex Misophonia Scale for Adults*

**DOI:** 10.1177/10731911241234104

**Published:** 2024-02-27

**Authors:** Julia Simner, Louisa J. Rinaldi, Jamie Ward

**Affiliations:** 1 University of Sussex, Brighton, UK

**Keywords:** misophonia, sound sensitivity, sensory sensitivity, aversion, assessment, validation, factor structure

## Abstract

Misophonia is a sound sensitivity disorder characterized by a strong aversion to specific sounds (e.g., chewing). Here we present the *Sussex Misophonia Scale for Adults* (*SMS-Adult*), within an online open-access portal, with automated scoring and results that can be shared ethically with users and professionals. Receiver operator characteristics show our questionnaire to be “excellent” and “good-to-excellent” at classifying misophonia, both when dividing our *n* = 501 adult participants by recruitment stream (self-declared misophonics vs. general population), and again when dividing them with by a prior measure of misophonia (as misophonics vs. non-misophonics). Factor analyses identified a five-factor structure in our 39 Likert-type items, and these were *Feelings/Isolation*, *Life consequences*, *Intersocial reactivity*, *Avoidance/Repulsion*, and *Pain*. Our measure also elicits misophonia triggers, each rated for their commonness in misophonia. We offer our open-access online tool for wider use (www.misophonia-hub.org), embedded within a well-stocked library of resources for misophonics, researchers, and clinicians.

Misophonia is a sound sensitivity disorder in which certain classes of sound feel unusually unpleasant (Swedo et al., 2022). Typical triggers are often human bodily sounds such as chewing, breathing, or lip-smacking. These sounds are easily ignored by most other people, but can be highly aversive to people with misophonia, causing negative emotions, such as anger, disgust, or anxiety, and sometimes a physiological reflex, such as increased muscle tension (Dozier & Morrison, 2017). Other triggers can be nonhuman noises, such as clicking or tapping, or even nonauditory repetitive actions, such as leg rocking.^1^ Misophonia symptoms have been linked to subtle organizational differences in the brain, including increased functional and structural connectivity in regions related to threat, emotion, and salience (Kumar et al., 2017; Schröder et al., 2019). This suggests sounds may have a larger salient and emotional impact for people with misophonia, compared with most other people.

Misophonia was first recognized by audiologist Marsha Johnson in 1997 (Bernstein et al., 2013) and named at the turn of the 21st century by M. M. Jastreboff and Jastreboff (2001). Early studies suggested as many as 20% of people report some degree of misophonia which can impact on daily life (e.g., Wu et al., 2014) with yet higher rates in groups with elevated anxiety (Naylor et al., 2021) but lower rates using other populations and measures (e.g., 5% prevalence in a German population; Jakubovski et al., 2022). However, it is difficult to draw a line between everyday disliking and the type of disliking found in misophonia. Many people find certain misophonia triggers at least somewhat unpleasant (e.g., messy slurping; Simner et al., 2021), but only people with clinically significant misophonia feel the anger or disgust that can make tolerating such sounds almost impossible.^2^ Our challenge as researchers is to design questionnaires with appropriate and validated diagnostic thresholds. This is one aim of the current article, where we present our *Sussex Misophonia Scale for Adults* (*SMS-Adult*) and demonstrate its ability to identify people with clinically significant misophonia successfully. We describe our platform and instrument below, alongside our exploratory factor analysis (EFA) and validations, which show our instrument to be “excellent” or for identifying misophonia (and a “good-to-excellent” match with an earlier measure).

One unique quality of our tool is that we created our adult questionnaire in such a way as to be ideally suited to adapting into a parallel questionnaire for children/adolescents, because misophonia is often reported from childhood (Claiborn et al., 2020; Rouw & Erfanian, 2018). We, therefore, ensured its language was concise and psycholinguistically appropriate for both adults and adolescents, and use stepwise conditional logic so that only positive responses require further detail (see Method). Our adolescent version of this adult misophonia scale has now been published with a validation of its scores based on children 10+ years. Hence, our adult questionnaire presented here is part of a family of instruments, which can provide valuable continuity of assessment across older and younger participants.

A second unique quality of our questionnaire is that we have packaged our instrument as a free online interactive assessment, embedded in an open-access testing platform with automated scoring, and a results portal to share data ethically between misophonics and professionals. Its feedback for participants includes whether the user passed the threshold, and what their problematic sounds appear to be. This feedback for participants is largely descriptive (i.e., it avoids being definitively diagnostic) and links to advice and support for misophonia, as well as to established routes for clinical support. Its feedback for professionals is more detailed, with item-by-item scoring. Results are presented both onscreen and as a downloadable csv file, making test data easily shared without the need for us to store identifying information. Importantly, results are delivered ethically to the participant themselves, but with clear signposting for how to share with researchers/clinicians (see the appendix).

Below, we report results from administering our tool on a large sample of adults with and without misophonia, classified in two different ways: first by recruitment streams (self-declared misophonics vs. a general population sample), and again using a separate independent measure of misophonia (the Misophonia Questionnaire [MQ]; Wu et al., 2014). In both cases, we use receiver operator characteristics (ROC) to show that our questionnaire is statistically “excellent” or “good-to-excellent” for classifying those with and without clinically significant misophonia.

Finally, to add further confidence in our measure, we point out that its scores have also been validated in three additional published papers, in both adults (Rinaldi et al., 2023; Simner et al., 2021) and adolescents (Rinaldi et al., 2022). These papers have shown that misophonics identified by our SMS questionnaire (using the thresholds we report here) also show a series of traits known to be *associated* with misophonia, namely, greater obsessive compulsive traits, anxiety (Cusack et al., 2018), autistic traits (Haq et al., 2020; Webber et al., 2014; Williams et al., 2021), attention-to-detail (Eijsker et al., 2021; Schröder et al., 2014), emotion dysregulation (Cassiello-Robbins et al., 2020), and poorer quality of life (Jaeger et al., 2020). Finally, these published papers also show that the scores from our measure do not converge with traits *un*related to misophonia (e.g., creative self-concept; Rinaldi et al., 2022). As such, the current article adds to this body of work, by providing new validations showing its ability to separate misophonics from non-misophonics, and its factor structure.

## Method

### Participants

In total, we tested 501 adults (*M*_age_ = 25.27, *SD* = 13.96), including 398 females (*M*_age_ = 25. 34, *SD* = 14.29), 82 males (*M*_age_ = 25.30, *SD* = 13.24), 12 nonbinary (*M*_age_ = 26.50, *SD* = 11.26), and nine who opted out of reporting their demographics. Of these, 143 were self-declared misophonics recruited from online misophonia communities (e.g., Facebook, Twitter; 103 females, *M*_age_ = 36.19, *SD* = 19.61; 29 males, *M*_age_ = 35.55, *SD* = 16.24; 7 nonbinary, *M*_age_ = 29.71 *SD* = 14.12; and four did not report demographics). The remaining 358 were recruited from the general population and local student population (295 females, *M*_age_ = 21.58, *SD* = 9.36; 53 males, *M*_age_ = 19.70, *SD* = 6.34; five nonbinary, *M*_age_ = 22.00 *SD* = 2.55; five did not report demographics). The latter took part in exchange for course credit. There was no self-selection bias for this comparison group, and they did not know the topic of the study before deciding to participate. Our study had ethical approval and was not preregistered.

### Materials and Procedure

Participants completed our study remotely, using our in-house testing platform, the *Misophonia Hub*, described in more detail further below. Participants were sent a URL via email to take part, and the study began with ethical consents. Participants then began our testing, which took 15-20 minutes, and consisted of our two target questionnaires shown in the order below.

### Sussex Misophonia Scale for Adults

#### Scale development

Our questionnaire for misophonia has two sections. In Part 1, participants were shown a series of triggers for misophonia, with the following question: *We’re going to ask you about things you see and hear every day. Have you always hated these things? Or don’t you mind them?* Then followed eight broad categories of trigger (e.g., *I hate . . . the sound of people eating*; see Table 1), which encapsulated every type of trigger we could gather from all relevant sources available at the time of testing (e.g., literature, firsthand reports). The only exception being that we avoided sounds (e.g., alarm, siren) that were possible triggers of hyperacusis, a separate condition in which everyday environmental sounds feel overwhelmingly loud or intense (Fackrell et al., 2017). Table 1 shows that seven out of our eight trigger categories were for sounds, and one was nonauditory because misophonia can also be triggered by repetitive visual movements such as leg-swaying (although this is reported less commonly so represented just one out of eight trigger categories). Participants responded Yes/No for each category of trigger, and if all eight responses were No, participants proceeded to Part 2. However, responding Yes for any category then revealed a full list of triggers *within* that category. For example, if participants responded Yes to *I hate the sound of people eating*, this revealed eight types of eating-sound (*crunchy foods*, *crispy snacks*, *chewing*, *lip-smacking*, *swallowing*, *slurping*, *wet mouth sounds*, *other eating sound*; see Table 1). The accompanying question was *Which do you hate hearing? Tick all that apply*. Across our eight categories, we presented 48 trigger items (see Table 1) using our conditional logic in a time-efficient way.

**Table 1.** Triggers for Misophonia and Their Superordinate Category.

No.	We’re going to ask you about things you see and hear every day. Have you always hated these things? Or don’t you mind them? I hate . . .	Which do you hate hearing (*or* seeing, *for Category 7*)? Tick all that apply.
1	The sound of people eating	Crunchy foods (e.g., apples), crispy snacks, chewing, lip-smacking, swallowing, slurping (a drink), wet mouth sounds (e.g., yogurt), other
2	The sound of repetitive tapping	Pen clicking, foot tapping/foot on floor, repetitive barking, tapping pen/pencil, tapping finger, typing on a computer, other
3	The sound of rustling	Rustling paper, rustling plastic, other
4	Throat sounds	Throat clearing, hiccups, humming, other
5	Sounds people make through their mouth and nose	Breathing, snorting (e.g., when people laugh), nose sniffing, coughing, snoring, whistling, sneezing, burping, other
6	Some voice sounds	Certain accents, some people’s voices, certain letter sounds, certain vowels, certain consonants, other
7	Repetitive visual movements	Repetitive leg rocking, foot shuffling, people rocking back and forth on their chair, other
8	Some background sounds (e.g., fridge humming)	Clock ticking, car engines, refrigerator humming, dishwasher, washing machine/dryer, fan, other

*Note.* Categories are shown first, and subset items revealed in the event of a positive response.

At the end of this section, participants passed automatically to Part 2, which presented (an original list of) 53 Likert-type statements (see Table 2).^3^ To generate these initial 53 items, we examined all items from all existing questionnaires identified by our literature review at the time of study (see Table 1—Supplementary Information) to determine recurrent themes. We additionally read firsthand reports from misophonics (e.g., from case studies and discussion forums online) to research further the range of themes and symptoms noted. We then wrote our initial 53 questionnaire items with an eye on clarity, simplicity, and age of acquisition (Gilhooly & Logie, 1980). The last was to ensure our adult questionnaire would be suitable for creating a parallel adolescent measure with only minimal adaptation (ultimately, it would require only a one-word substitution *work*→*school* in four items; e.g., *I avoid work*→*I avoid school*; see below and our adolescent version in Rinaldi et al. (2022)). Statements loosely fell into a number of different themes, for example, (a) emotions (e.g., *Sounds that other people don’t mind can make me really angry*); (b) behaviors (real or imagined), including both reactive behaviors (e.g., *I want to hurt people who make sounds I hate*) and avoidant behaviors (e.g., *I cover my ears to block out certain sounds*); and (c) consequences in other areas of life such as in relationships or work (e.g., *My hatred of some sounds creates problems in work*).^4^ For each statement, participants chose their rating on a 5-point scale (*Never*, *Hardly ever*, *Sometimes*, *Often*, *Always*).

**Table 2.** Five-Factor Model Loadings.

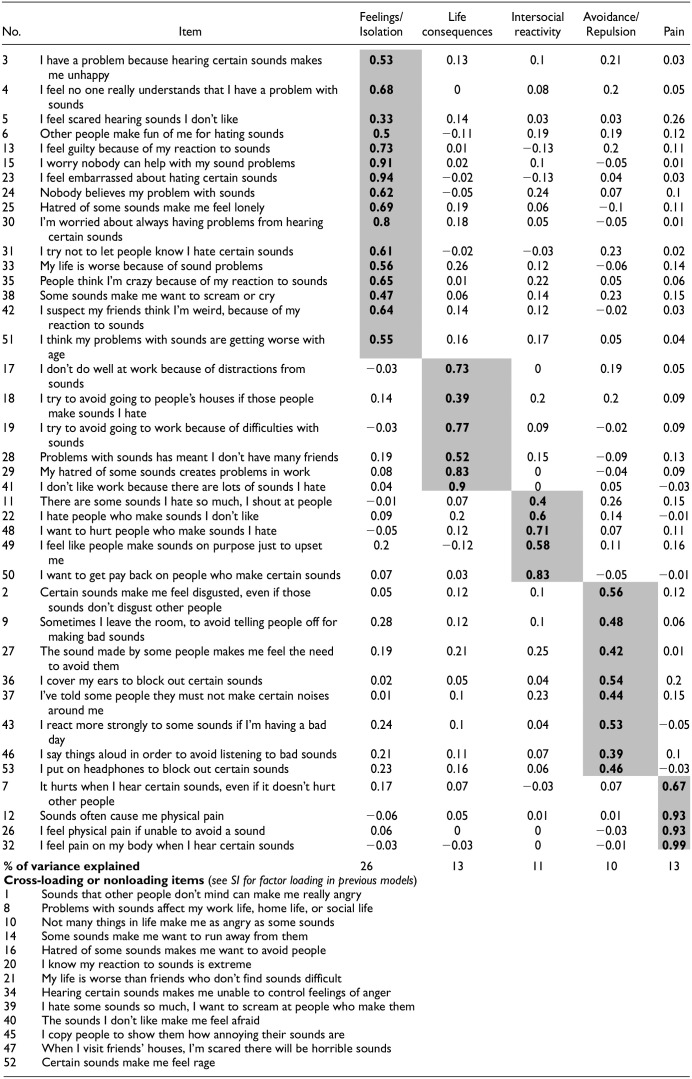

*Note.* Table shows all items in Part 2 of our measure (Likert-type items) and their factor loadings. Bold represents factor loadings >3.00, within one of five factors, named in the table header. Bottom of table shows items that did not satisfy our criteria for any factor. Item numbers (No.) represent ordering within our original questionnaire. Previous iterations of this factor analysis, including rejected cross-loading items, are found in Table 2 of the SI.

We also included items from a theme related to pain (e.g., *Sounds often cause me physical pain*), which we had also identified from previous misophonia literature and firsthand reports. Some prior studies have clearly included pain in their diagnostic criteria for misophonia (e.g., Dozier et al., 2017), whereas others have not (Swedo et al., 2022). It is also possible that people with misophonia experience pain via comorbid conditions such as hyperacusis (Tyler et al., 2014, see also Fackrell et al., 2017) or tensor tympani syndrome (Westcott, 2016). Here, we remain agnostic as to the role of pain in the definition of misophonia, but included pain (a) because people with misophonia report pain (e.g., Claiborn et al., 2020; Dozier, 2015; Edelstein et al., 2013) whether that be directly or via known comorbidities (e.g., 69.9% of people with hyperacusis also had misophonia; P. J. Jastreboff & Jastreboff, 2014); (b) for its clinical usefulness, allowing clinicians diagnosing misophonia to also observe whether pain is an accompanying factor; and (c) because pain is difficult to measure in any other way.

In total, Parts 1 and 2 of our questionnaire contained 109 items (in long form), with 48 items revealed only conditionally, so our questionnaire took just 10 minutes to complete.

#### The Automated Interface: www.misophonia-hub.org

**Figure A1.**
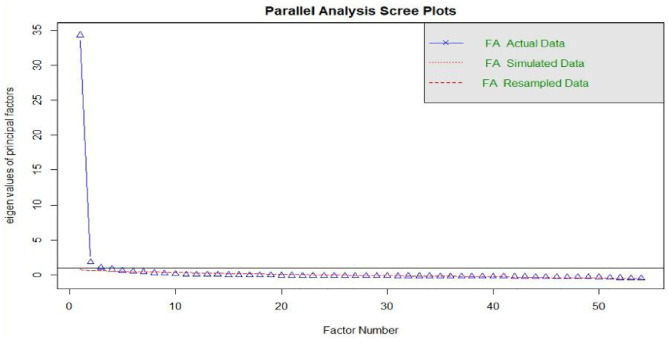
Automatic Results Output Screens From the SMS-A on the Misophonia Hub (www.misophonia-hub.org). *Note.* The examples show a pass at both Parts 1 and 2. Top shows first results screen designed to give clear concise results for participant. Bottom shows second results screen designed to give more detailed item-by-item results for researchers/clinicians.

We housed the *SMS-Adult* within the *Misophonia Hub*, our purpose-built interactive online platform for testing and supporting misophonia (www.misophonia-hub.org). In this platform, participants ethically consent to the study and complete the questionnaire using interactive on-screen buttons. At the end of the test, it delivers automated scoring and two types of results feedback. The first is for participants (see the appendix, Figure A1, top panel) and the second is a more detailed screen with an item-by-item breakdown of results, a downloadable csv file, and a codebook (see Figure A1, bottom panel). We produced these latter, more detailed, results with professionals in mind, and placed them within a clearly defined box. This box is colored orange, and the clear flagging with color and shape allows a researcher/clinician to test someone remotely, and simply request “the results at the end in the orange box” (or indeed the clearly marked downloadable csv file).

Regarding ethics, our portal has been approved for wider use by the Sussex University Science and Technology Ethics Committee (i.e., for use by any incoming user, sent by any researcher/clinician; with its ethics application—approval available on request from the first author [J.S.]). Participants do not provide their personal details to take their test, and results are delivered directly to the participant (who can subsequently pass these onto a third-party researcher/clinician if desired). Test data enter our database anonymously and are only linked to identifying information if the participant later wishes subsequently to join our own participant database. In other words, after the test is finished and results delivered, the participant can leave—but our final screen is also an invitation to join our testing group (which can be ignored). Participants are under no obligation to do this and still have the full functionality of the test/results as an anonymous user without this.

Alongside this testing portal, the Misophonia Hub was also programmed with four additional portals. Two provide support and information about misophonia for adults and children/parents/teachers, respectively. These include downloadable misophonia factsheets which can be personalized, then shared with a clinician, teacher, or other relevant third party. Two final portals contain information for researchers and clinicians, respectively, including, for example, a science bibliography and review of published therapeutic approaches. As such, we designed our website not only to host our test for future users (e.g., clinicians measuring intake severity and ongoing response to treatment), but also to provide a support library for the misophonics and professionals who will use it.

### The Misophonia Questionnaire

The MQ (Wu et al., 2014) is the most widely cited questionnaire in the misophonia literature and contains 21 questions across three sections. Sections 1 and 2 contain items on triggers (*n* = 8) and emotions/behaviors (*n* = 11), respectively; both are answered using a 5-point scale (0—*Not at all true* to 4—*Always true*) and show good internal consistency (α = .86). Section 3 is a 15-point severity scale, where participants assess their own severity by taking into account their number of triggers, degree of distress, and impairment in their lives. Individuals reporting ≥7 on this Severity Scale are considered to have clinically significant misophonia (Wu et al., 2014).

## Results

### Approach to Analysis

We first explore the factor structure of our questionnaire, then validate its scores and establish a threshold by considering its ROC. Analyses were performed in R Version 3.6.3 (R Core Team, 2022) using R Studio; we used *tidyverse* for general data wrangling (Wickham et al., 2019), *psych* to perform EFA and parallel analysis (Revelle, 2022), *Hmisc* for correlations (Harrell & Dupont, 2020), and *pROC* to produce ROC plots (Robin et al., 2011). The assumption of normality was tested by exploring histograms and QQ plots which showed acceptable near-normality (with a slightly elongated upper tail as expected, because most people do not have misophonia). Assumptions of correlation and sampling adequacy were tested and supported using Bartlett’s test of correlation adequacy, X^2^(1,431) = 32,288.85, *p* < .001 (Bartlett, 1951), and Kaiser–Meyer–Olkin (KMO) showed sampling adequacy *MSA =* 0.98 (Kaiser, 1960).

### Exploratory Factor Analysis

**Figure 1.**
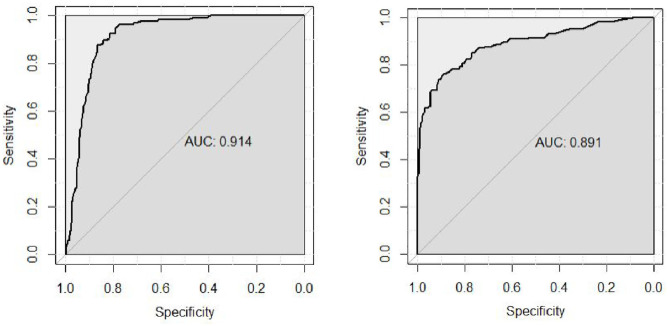
ROC Curves Showing the Sensitivity of Our Test Plotted Against Specificity. *Note. Left plot* shows our test to be an “excellent” predictor of group status (misophonics vs. controls) using recruitment streams. *Right plot* shows our test to be a “good-to-excellent” predictor of group status using the MQ threshold (Wu et al., 2014).

We first analyzed the underlying factors within Part 2 of our scale (our Likert-type items). We used multiple strands of evidence to determine that a five-factor solution would be optimal. We first ran a parallel analysis (Horn, 1965) which compared our data with simulated data to determine the number of factors appearing at greater than chance. This indicated five factors. Our EFA then placed the maximum number of factors at five, with five factors meeting the Kaiser criterion (eigenvalues ≥1, i.e., 34.45, 2.23, 1.43, 1.24, and 1.03; Kaiser, 1960; but see Russell, 2002). Per the parallel analysis, our scree plot showed the five factors above the chance line from simulated data, although its elbow raised the possibility of a three-factor model (see Figure 1—SI). We, therefore, explored both three- and four-factor models also. The four-factor solution failed to achieve simple structure after five rounds of removing cross-loading items and had only one item on the fourth factor that did not cross-load. The three-factor model included a larger number of items (*n* = 49), but with most falling into an incoherent factor (39 items) mixing work, behaviors, emotions, friendship, and so on. In contrast, the five-factor model achieved simple structure with five coherent categories, which we established as follows.

As noted, we proceeded to extract five factors in our EFA using maximum likelihood estimation, and direct oblimin rotation, because we expected that factors would correlate (Clarkson & Jennrich, 1988). Following (Preacher & MacCallum, 2003), we wanted to ensure each of our items loaded onto at least one factor—and only one factor— greater than .30. Our initial analyses of all 53 items showed 11 items did not meet these criteria. We excluded these items (8, 10, 14, 20, 21, 34, 39, 40, 45, 47, 52; see Table 2), then repeated the process to remove a further two (1, 16; full factor solutions for these prior iterations are presented in Tables 2 and 3 in our SI). We next re-ran our EFA, and our resulting model achieved simple structure, which is to say that each item loaded only onto one factor, greater than .30. These factor loadings are shown in Table 2. Our model had overall acceptable-to-excellent fit on multiple measures: the root mean square of the residuals (RMSR) indicated “excellent” fit at 0.02, the root mean square error of approximation (RMSEA) index indicated “acceptable” fit at 0.07 (confidence intervals [CIs] = [0.067, 0.074]), and both the comparative fit index (CFI) and Tucker–Lewis index (TLI) were “acceptable” and just short of the “excellent” threshold, at CFI = 0.94 and TLI = 0.92, respectively (Buchanan, 2020). Finally, we evaluated the most marginal factor of the five factors from the scree plot, to confirm its psychological interpretability. This showed itself to be a unified factor comprising items relating to pain (e.g., *Sounds often cause me physical pain*), which has a highly useful function in MQs. People with misophonia report pain as a distinct experience (see above; e.g., Claiborn et al., 2020; Dozier, 2015; Edelstein et al., 2013), and a pain factor is clinically useful in allowing clinicians also to observe whether pain is an accompanying symptom. On the balance of this evidence, we therefore extracted five factors from our questionnaire, and these are described below.

**Table 3.** Correlation Matrix Showing Reliability Between Scores on the Sussex Misophonia Scale and the MQ, With Values Above the Diagonal (of 1s) Displaying Correlations for Controls, and Values Below the Diagonal Displaying Correlations for Misophonics.

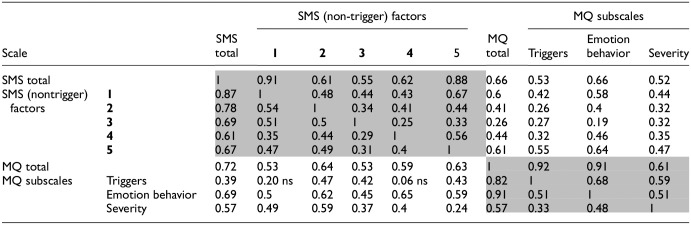

*Note.* Table shows total scores on each subscale, as well as the SMS factors (1 = Feelings/Isolation; 2 = Life consequences; 3 = Intersocial reactivity; 4 = Avoidance/Repulsion; 5 = Pain) and MQ subscales. Note that the MQ subscale here called “triggers” is named “symptoms” at source (Wu et al., 2014) and that no SMS factors relate to triggers. This explains the lower correlations between SMS factors and the MQ triggers subscale. All other correlations are high, both within our own subscales (dark gray shading) and between measures (no shading). Light gray shadings show our data for correlations within the MQ. SMS = Sussex Misophonia Scale; MQ = Misophonia Questionnaire. All correlations are significant at *p* <.001 except where otherwise shown.

In sum, our final questionnaire (see the appendix) contained 39 Likert-type items (as well as the 48 trigger items in Part 1), with the following five factors. Factor 1 consisted of 16 items relating to *Feelings and Isolation*, with items such as *Hatred of some sounds make me feel lonely*. Factor 2 consisted of six items which describe *Life consequences* (i.e., impact on work and friendships), including, for example, *I don’t do well at work because of distractions from sounds*. Factor 3 consisted of five items which relate to *Intersocial reactivity* with items such as *I want to get pay back on people who make certain sounds*. Factor 4 consisted of eight items which deal with *Avoidance/Repulsion* with items such as *I cover my ears to block out certain sounds*. Finally, Factor 5 consisted of our items related to *Pain*; it contained four items such as *I feel physical pain if unable to avoid a sound*. Internal reliability of all factors was very high with Cronbach’s alpha estimates (Cronbach, 1951) of .98, .94, .91, .92, and .95 for Factors 1 to 5, respectively. Our Factor 1 has a slightly elevated alpha (beyond the recommended .95 for clinical tests; Bland & Altman, 1997), which means several items may be redundant, making our test marginally longer than it needed to be. We have, therefore, added factor correlations in Table 4—SI to show there are no concerns about collinearity (i.e., all below .80; Field et al., 2012).

### Establishing a Cutoff Threshold to Identify Misophonia: ROC Analysis

One aim of our research is to establish a statistically valid cutoff to identify people with clinically significant misophonia. To achieve this, we calculated the total score across all five factors in our final *SMS-Adult* detailed above (i.e., 39 items), where responses were coded 0 to 4 (from *Never* to *Always*), and scores ran from 0 to 156. We then subjected participants’ total scores to an ROC analysis, to predict whether individuals were from the self-declared misophonia group or general population comparison group. This analysis returns an area under the curve (AUC) which runs from .5 (chance prediction) to 1 (perfect predictive classification). The AUC from our data was .91 (95% CI = [.88, .94]; see Figure 1, left panel), suggesting our test has “excellent” classification accuracy (Mehdi & Ahmadi, 2011). We then selected a threshold to maximize both sensitivity (true-positive rate) and specificity (true-negative rate) using the *Youden* method (Hughes, 2015; Youden, 1950). This established a cutoff score threshold for misophonia at 50.5 (out of 156), which successfully identifies 87.5% of misophonics while excluding 86.2% of controls (see Figure 1 [left] for the ROC plot). This statistical threshold (50.5) was programmed into our final online test at www.misophonia-hub.org/test for future users. In our discussion, we address what it means to have a threshold score for a condition with a broad spectrum of severity ranging from annoying to debilitating, and how to interpret our threshold given our selected samples.

Using our new threshold for misophonia, we next re-categorized our participants into a group we call *SMS-Misophonics* (i.e., those confirmed as misophonic by scoring above threshold in the *SMS-Adult; n*=165, 123 female, 29 male, nine nonbinary, four did not report gender) and a group called *SMS-Non-Misophonics* (i.e., those scoring *below* threshold; *n* = 301, 241 female, 52 male, three nonbinary, five did not report gender).^5^ Table A1 in our appendix shows their descriptive statistics (means (*M*), standard deviations [*SD*s], and 95% CIs, for the overall score, and within each factor). We also include a second table (Table A2) where we repeat these data but now consider only participants recruited from our general population stream (to provide descriptive statistics from a community sample). As expected, these statistics are largely equivalent across Tables A1 and A2, with corrected *p*s greater than .803 (and uncorrected *p*s falling between .134 and .883).

### Validity Against an Existing Misophonia Measure

We next measured validity against an existing MQ which participants also completed (MQ; Wu et al., 2014). We analyzed these data in two ways. First, we ran a correlation matrix between all elements of the Sussex Misophonia Scale (i.e., total score Part 2 and its five factors) against the MQ (total score and its three subscales). The resulting correlation matrix (Table 3) shows significant moderate-to-high correlations, not only within the Sussex Misophonia Scale (Table 3, dark gray shading), and within the MQ (light gray shading), but also across measures (no shading). As expected, smaller correlations (small-to-moderate, i.e., *r* = 0.20–0.55) were found between our measure and the MQ’s Symptoms subscale (named “triggers” in our table, for clarity). This smaller correlation is exactly as expected because our own data (total score Part 2, plus factors) are based on emotions, behaviors, and outcomes, whereas this particular MQ scale is based on triggers (which are within our Part 1, but not amenable to this type of analysis).

We also used the MQ to verify, in a second way, the statistical value of our own scale. Thus far, we have categorized participants according to their recruitment stream (self-referred misophonics vs. comparison group from the general population) or by their scores on our Sussex Misophonia Scale (*SMS-Misophonics* vs. *SMS-Non-Misophonics)*. Below, however, we begin again entirely agnostic to their status or recruitment streams, but instead assign participants to groups using their MQ scores and its published diagnostic threshold.^6^ Once our groups were re-defined, we had 198 MQ-defined misophonics (159 female/26 male/seven nonbinary/six preferred not to say; *M*_age_ = 30.03, *SD* = 17.37) and 288 MQ-defined non-misophonics (225 female/55 male/five nonbinary/three preferred not to say; *M*_age_ = 22.16, *SD* = 9.67). We then re-ran our ROC analysis and found that our test was again a highly robust measure for identifying misophonics. Our AUC was now .89 (95% CI = [.86, .92]) meaning our test is considered “good-to-excellent” (Mehdi & Ahmadi, 2011). See Figure 1 (right panel) for the ROC plot. This analysis adds additional validity for the scores from our measure.

### Analyzing Misophonia Triggers

Thus far, we have considered Part 2 of our Sussex Misophonia Scale (Likert-type responses for emotions/behaviors/outcomes, etc.). Here, we turn to Part 1 (triggers), and first demonstrate, as expected, that people classified as misophonic report more triggers (e.g., chewing) and a wider range of categories than people without misophonia. Our two groups were again SMS-Misophonics and Non-Misophonics (i.e., using our Sussex Misophonia Scale Part 2 threshold of 50.5; see above). SMS-Misophonics had, on average, 16.52 triggers (*SD* = 7.68) across 5.40 categories (*SD* = 1.88), whereas non-misophonic controls had 6.52^7^ (*SD* = 5.81) and 2.79 (*SD* = 1.91), respectively. This difference was significant in a Welch independent-samples *t* test for both items, *t*(268.49) = −14.59, *p*< .001, and categories, *t*(342.32) = −14.18, *p*< .001, which adds validation to our measure’s threshold.

Finally, we consider how our data on triggers (Part 1) might be useful in diagnostic terms. Here, we ranked triggers from highest (most common among our SMS-Misophonics) to lowest (see the appendix; also Table 5—SI). Our proposal is that clinically significant misophonia can be identified not only by scoring above threshold in Part 2 (on feelings/behaviors/outcomes, etc., of misophonia), but as also having typical misophonia triggers (i.e., at least one trigger within the top *n* aversive triggers experienced by misophonics). We determine that 99.4% of misophonics had at least one trigger from our top 39 triggers (see Table 5—SI). Hence, setting a boundary at the 39th most common trigger means we can capture 99.4% of the individuals with misophonics in our sample (but this cutoff would benefit from cross-validation in a future independent sample). We discuss this boundary in more detail in our discussion.

## Discussion

In this article, we presented our novel *SMS-Adult*. This is shown in final version in our appendix and is available for free open-access use with automated scoring at www.misophonia-hub.org/test. Below, we discuss our validation and factor analyses in turn.

### Validation and Diagnostic Criteria for Researchers and Clinicians

Our final *SMS-Adult* is a two-part questionnaire, where Part 1 presents misophonia triggers (48 triggers within eight different categories), and Part 2 determines the feelings, behaviors, and outcomes associated with misophonia (39 Likert-type questions). Our ROC analysis on Part 2 suggests that the Sussex Misophonia Scale is an “excellent” measure to distinguish people with misophonia from a general population comparison group. Our analyses show that we gain maximum sensitivity and specificity with a threshold score of 50.5 or higher (out of 156; in Part 2) which captures 88% of misophonics while excluding 86% of controls. These values are clearly within the principles “for high-quality, high-value testing” from the *British Medical Journal* (Power et al., 2013, p. 6) where [% sensitivity + % specificity] should be greater than 150 (ours is 174^8^). Finally, we provided support for our test a second time, now dividing our participants using an independent assessment of misophonia (MQ; Wu et al., 2014). Again our ROC analysis provided support for our *SMS-Adult* questionnaire, showing it to be a “good-to-excellent” predictor of misophonia (as classified by the MQ).

We should also consider what our threshold means in relation to whom we studied. A key limitation of all medical and psychological tests is how to establish the objective truth of who does and does not have the condition of interest (here, misophonia). In our case, the threshold we provide (50.5) distinguishes a group of self-referred misophonics from a general population sample, so we must interpret our threshold in that context. Our 86% specificity means 14% of our general population sample were considered to have misophonia. However, 14% fits within the range of expected prevalence for misophonia (Jakubovski et al., 2022; Wu et al., 2014; Zhou et al., 2017), suggesting we may have a yet more accurate test than it seemed at first glance. In other words, this 14% may not be an error rate at all; it might merely indicate that our test is exceptionally accurate and was therefore correctly identifying the *true* misophonics in the general population sample (at around the prevalence we would expect).

Our sensitivity of 88% means our test was not sensitive enough to detect 12% of self-referred misophonics, who were—by definition—those reporting less severe symptoms. But this sensitivity is compatible with the accuracy of a swathe of other medical tests (e.g., for gestational diabetes = 85%; sarcoidosis = 73%; Donovan et al., 2013; Parikh et al., 2008; for review across multiple conditions, see Maxim et al., 2014). Finally, we note that we have set a threshold because it is the purpose of any “diagnostic” test to do so. Although our test is highly successful (i.e., “excellent” in terms of its validation scores and threshold), we point out that misophonia remains a condition with a broad spectrum of severity ranging from annoying to debilitating. Our threshold should therefore be considered as identifying a clinically significant level of misophonia (i.e., involving negative feelings, isolation, problems at work, etc.).

Our Part 1 data allowed us to rank misophonia triggers from most to least common, according to how often they were experienced by our confirmed misophonia group. We found that 99.4% of our misophonics had at least one trigger within the top 39. This increases confidence in our scale because some MQs risk capturing people whose lives are affected by “a sound sensitivity,” whether this is misophonia or not. Consider, for example, the Severity Scale of the MQ, which instructs participants to *Please indicate the severity of your sound sensitivity* . . . and so arguably applies to any sound sensitivity (e.g., misophonia, hyperacusis). The same is true of many other questionnaires, and indeed certain items within our own scale (e.g., *I have a problem because hearing certain sounds makes me unhappy*). To our knowledge, we are the first to point out this potentially problematic issue. However, we suggest our (Part 2) questionnaire captures misophonia itself, for several reasons. First, a number of questions are specific to misophonia, and misophonia only (e.g., Factor 4: *Certain sounds make me feel disgusted, even if those sounds don’t disgust other people*), and this factor correlates highly with others (at .76 > *r* > .87*)*. Second, we found support for our scale from self-declared misophonics, suggesting our ROC is indicating success in this domain. Finally, we can be confident our scale recognizes misophonia because 99.4% of our misophonics reported a known misophonia trigger. We, therefore, encourage users of our test to consider whether an above-threshold score in Part 2 co-occurs with at least one misophonia trigger in Part 1.

We note that 99.4% of our misophonics had a trigger within the top 39 ranked triggers for misophonia, so one option might be to impose this as part of the diagnostic criteria (i.e., a score higher than threshold [in Part 2] *plus* at least one trigger within the top 39 [in Part 1]). However, we instead suggest a more agnostic standard, to avoid dismissing misophonics with rare/unknown triggers (or indeed, anyone who skipped Part 1 to avoid thinking about their triggers). Hence, our final diagnostic criterion is to pass the ROC-validated threshold of 50.5 (out of 156) in Part 2, but with Part 1 as a descriptive statistic. Our final test and thresholds are shown in the appendix and are available at www.misophonia-hub.org/test, where scores and feedback are provided automatically at the end of the test (see the appendix for feedback protocols).

### Factor Structure Within Misophonia: A Five-Factor Solution

Our analyses also revealed an underlying five-factor structure in Part 2 of our questionnaire, as follows: *Feelings & Isolation* (having misophonia feels bad and increases isolation); *Avoidance/Repulsion* (actively avoiding sounds or being repulsed/disgusted by them), *Intersocial reactivity* (negative feelings toward others who make sounds), *Life consequences* (impacting negatively on work or friendships), and *Pain* (sounds cause physical pain).

Our factors share commonalities with those from three questionnaires created at the same time as our own, which have since entered the literature (Dibb et al., 2021; Siepsiak, Śliwerski, et al., 2020; Vitoratou et al., 2021). Our *Feelings/Isolation subscale* has elements in common with subscales named *Affective Response* and *Beliefs* (Rosenthal et al., 2021), *Threat* (Vitoratou et al., 2021), and *Emotional* (Dibb et al., 2021). Similarly, our *Avoidance/Repulsion* subscale shares features with *Cognitive* and *Coping scales* (Rosenthal et al., 2021), while our *Intersocial reactivity* shares features with *Behavioral Responses* (Rosenthal et al., 2021) *Externalizing* and *Outbursts* (Vitoratou et al., 2021). Our *Life consequences* shows commonalities with subscales named *Impairment* (Rosenthal et al., 2021), *Impact* (Vitoratou et al., 2021), and *Participation* (Dibb et al., 2021), and our *Pain* factor with subscales called *Physiological* (Dibb et al., 2021) and *Physical Responses* (Rosenthal et al., 2021). This commonality provides additional support for the five factors of our questionnaires and, overall, provides considerable evidence that misophonia is a complex trait, with multiple subfacets.

#### Summary and Limitations

Although our questionnaire is relatively comprehensive, certain facets were not explored. One area not examined here is captured elsewhere by the *Internalizing* subscale from the S-Five Questionnaire, that is, placing blame internally (Vitoratou et al., 2021). We, therefore, recommend this latter questionnaire for those interested in self-blame (while our own questionnaire has scales beyond the S-Five, of *Avoidance/Repulsion* and *Pain*). Similarly, the physiological facets of misophonia are covered here only minimally, by our limited *Pain* factor. We, therefore, point the reader to the excellent work of Dozier and colleagues (e.g., Bauman & Dozier, 2013; Dozier, 2015) and suggest the user would be best placed to apply Dozier’s extensive physiological questionnaires when interested in the physiological elements of misophonia in particular. Likewise, for those interested in *nonauditory* misophonia (e.g., triggered by visual stimuli such as leg rocking), our own questionnaire explored this in Part 1 only. We, therefore, recommend measures such as the Misophonia Assessment Questionnaire (Johnson, 2014, revised by Dozier, 2015) which does not use the word “sounds” in its Likert-type items (and so applies to both auditory and visual triggers).

A second limitation might be that our data are based on self-report and so further research will be useful to explore the full range of MQs with further objective means. A third limitation is that participants can endorse any one of our 48 triggers, but cannot indicate the intensity of their disliking (although we have been able to show which triggers are most disliked in terms of their commonness across multiple misophonics). A fourth limitation is that establishing a threshold belies the fact that the expression of misophonic symptoms varies, as does its severity, and can range from mild to severe. However, our approach in placing a threshold is the clinical and research standard, and applies across thousands of conditions, both physical and psychological. Our threshold of 50.5 reflects a reasonable degree of clinical difficulty. For example, someone with a score of 40 would not have clinically significant misophonia because any problems with sound simply “hardly ever happen” (i.e., for 38 out of 39 items). In contrast, passing the 50.5 threshold means that almost half the problems are happening “often,” or perhaps that a full third are happening 100% of the time (e.g., problems at work, limit to social life, isolation). This, therefore, represents the lower end of the clinically significant spectrum of misophonia, as well as being the statistically “excellent” threshold in our ROC statistics.

In terms of our threshold setting itself, it could be argued that our sampling of people with misophonia from online communities might recruit more severe cases than we would find in the general population at large. However, much current research examines a *yet more severe* population, which are individuals with misophonia who have sought clinical support (Cassiello-Robbins et al., 2020; Jaeger et al., 2020). By comparison, our own sample is likely to be more representative of typical misophonics in the general population. In addition, we remind the reader that our 86% specificity means that 14% of our general population controls were considered to have misophonia—which, crucially, fits within the range of expected prevalence for misophonia (Jakubovski et al., 2022; Wu et al., 2014; Zhou et al., 2017). In other words, setting our threshold at 50.5 according to recruitment streams in fact represents a highly accurate test, according to current estimates of prevalence.

We also point out that two thirds of our self-declared misophonics were female, which is to be expected in typical study-volunteer populations (Dindia & Allen, 1992), and has been a common feature of MQs previously (e.g., Dibb et al., 2021; Wu et al., 2014). Women with misophonia have sometimes shown quantitatively stronger misophonia in certain measures (Siepsiak, Sobczak, et al., 2020) though not in others (Vitoratou et al., 2021; Wu et al., 2014). Future studies might seek to understand better whether there is (or is not) a misophonia gender-phenotype. We also note that future work might seek to replicate our five-factor structure or seek validation beyond our ROC analyses here (e.g., in longitudinal consistency). However, three additional published validation studies now provide further support for our measure, in both adults (Rinaldi et al., 2023; Simner et al., 2021) and adolescents (Rinaldi et al., 2022). Finally, we point out that our MQ asks about disliking triggers (e.g., crunchy food) using the phrase “Have you always . . . .” This phrase is particularly useful for reducing false positives, which sometimes arise from suggestibility (i.e., sudden agreement during testing without any history of disliking sounds in the past). However, many misophonics may remember a time in earlier life before the onset of symptoms (Claiborn et al., 2020), raising questions over the usefulness of the word “always.” For now we note this limitation and hope future studies will better understand the balance between false positives and negatives in questioning misophonics about their experiences.

Our final limitation is that there may be a certain amount of redundancy in our items because inter-item correlations for our subscales were high. Nonetheless, we retained all 39 items for the following reasons: (a) Our ROC analysis shows our questionnaire is “excellent” in its current form for discriminating clinically significant misophonics from controls, and the number of items (*n* = 39) is not overly onerous; (b) our 39-item measure has been accompanied by three further validation papers providing additional validity (see above), and its current form has been able to provide a great deal of useful published information, albeit with the possibility of minor redundancy which we identify here; (c) retaining all 39 items brings the advantage of a continuity of assessment across the lifespan, because this adult scale has a published adolescent version designed very carefully to align with it (Rinaldi et al., 2022). It is therefore preferable to maintain this continuity of assessment by retaining what may be several redundant items, but yet transparently addressing this issue here. As such, future users can appreciate a full and complete picture of the measure without losing its benefits and prior history.

In summary, we have produced a concise questionnaire for misophonia, which we shown to have “excellent” (or “good-to-excellent”) properties in identifying people with misophonia. In Part 1, our measure elicits triggers for misophonia, and in Part 2, it elicits a five-factor structure of behaviors, feelings, and outcomes. Our final questionnaire (see the appendix and www.misophonia-hub.org/test) has a number of unique advantages alongside its validation and factor structure, having a parallel-published adolescent version for continuity of assessment, a free open-access online interface, and automatic scoring and feedback which is easily and ethically shared with researchers and clinicians.

## Supplemental Material

sj-docx-1-asm-10.1177_10731911241234104 – Supplemental material for An Automated Online Measure for Misophonia: The Sussex Misophonia Scale for Adults Supplemental material, sj-docx-1-asm-10.1177_10731911241234104 for An Automated Online Measure for Misophonia: The Sussex Misophonia Scale for Adults by Julia Simner, Louisa J. Rinaldi and Jamie Ward in Assessment

## Appendix

### The *Sussex Misophonia Scale for Adults* (*SMS-Adult*)

#### Part 1

We’re going to ask you about things you see and hear every day. Have you always hated these things? Or don’t you mind them? I hate . . . (*Categories 1–8 are shown on screen, with subscale items revealed in the event of a positive response to the category*)

1. The sound of people eating yes/no
  Which do you hate hearing? Tick all that apply.
  Crunchy foods (e.g., apples), crispy snacks, chewing, lip-smacking, swallowing, slurping (a drink), wet mouth sounds (e.g., yogurt), other
2. The sound of repetitive tapping yes/no
  Which do you hate hearing? Tick all that apply.
  Pen clicking, foot tapping/foot on floor, repetitive barking, tapping pen/pencil, tapping finger, typing on a computer, other
3. The sound of rustling yes/no
  Which do you hate hearing? Tick all that apply.
  Rustling paper, rustling plastic, other
4. Throat sounds yes/no
  Which do you hate hearing? Tick all that apply.
  Throat clearing, hiccups, humming, other
5. Sounds people make through their mouth and nose yes/no
  Which do you hate hearing? Tick all that apply.
  Breathing, snorting (e.g., when people laugh), nose sniffing, coughing, snoring, whistling, sneezing, burping, other
6. Some voice sounds yes/no
  Which do you hate hearing? Tick all that apply.
  Certain accents, some people’s voices, certain letter sounds, certain vowels, certain consonants, other
7. Repetitive visual movements yes/no
  Which do you hate seeing? Tick all that apply.
  Repetitive leg rocking, foot shuffling, people rocking back and forth on their chair, other
8. Some background sounds (e.g., fridge humming) yes/no
  Which do you hate hearing? Tick all that apply.
  Clock ticking, car engines, refrigerator humming, dishwasher, washing machine/dryer, fan, other

#### Part 2

How often do these things happen to you? (Likert-type responses: *Never*, *Hardly ever*, *Sometimes*, *Often*, *Always*).

1. Certain sounds make me feel disgusted, even if those sounds don’t disgust other people.
2. I have a problem because hearing certain sounds makes me unhappy.
3. I feel no one really understands that I have a problem with sounds.
4. I feel scared hearing sounds I don’t like.
5. Other people make fun of me for hating sounds.
6. It hurts when I hear certain sounds, even if it doesn’t hurt other people.
7. Sometimes I leave the room, to avoid telling people off for making bad sounds.
8. There are some sounds I hate so much, I shout at people.
9. Sounds often cause me physical pain.
10. I feel guilty because of my reaction to sounds.
11. I worry nobody can help with my sound problems.
12. I don’t do well at work because of distractions from sounds.
13. I try to avoid going to people’s houses if those people make sounds I hate.
14. I try to avoid going to work because of difficulties with sounds.
15. I hate people who make sounds I don’t like.
16. I feel embarrassed about hating certain sounds.
17. Nobody believes my problem with sounds.
18. Hatred of some sounds make me feel lonely.
19. I feel physical pain if unable to avoid a sound.
20. The sound made by some people makes me feel the need to avoid them.
21. Problems with sounds has meant I don’t have many friends.
22. My hatred of some sounds creates problems in work.
23. I’m worried about always having problems from hearing certain sounds.
24. I try not to let people know I hate certain sounds.
25. I feel pain on my body when I hear certain sounds.
26. My life is worse because of sound problems.
27. People think I’m crazy because of my reaction to sounds.
28. I cover my ears to block out certain sounds.
29. I’ve told some people they must not make certain noises around me.
30. Some sounds make me want to scream or cry.
31. I don’t like work because there are lots of sounds I hate.
32. I suspect my friends think I’m weird, because of my reaction to sounds.
33. I react more strongly to some sounds if I’m having a bad day.
34. I say things aloud to avoid listening to bad sounds.
35. I want to hurt people who make sounds I hate.
36. I feel like people make sounds on purpose just to upset me.
37. I want to get pay back on people who make certain sounds.
38. I think my problems with sounds are getting worse with age.
39. I put on headphones to block out certain sounds.

### Scoring

The passing criterion is based on scores in Part 2.

Part 2. Likert-type responses are scored 0 to 4 (0—*never*; 1—*hardly ever*; 2—*sometimes*; 3—*often*; 4—*always*) and are then summed to give scores running from 0 to 156, where the passing threshold is 50.5, meaning scores of 51 or higher indicate misophonia.

Factor 1 (Feelings/Isolation) items: 2, 3, 4, 5, 10, 11, 16, 17, 18, 23, 24, 26, 27, 30, 32, 38
Factor 2 (Life consequences) items: 12, 13, 14, 21, 22, 31
Factor 3 (Intersocial reactivity) items: 8, 15, 35, 36, 37
Factor 4 (Avoidance/Repulsion) items: 1, 7, 20, 28, 29, 33, 34, 39
Factor 5 (Pain) items: 6, 9, 19, 25

**Table A1.** Descriptive Statistics: Means, Standard Deviations (SD), and 95% Confidence Intervals for 165 SMS-Misophonics and 301 SMS-Non-Misophonics (Using Our Whole Sample), in Our Scale and Its Five Factors

Factor	*SMS-Adult*	Misophonic status (from *SMS-Adult*)	*M*	*SD*	Confidence interval
Overall	Total—Part 2	Misophonics	89.62	23.68	85.98-93.26
		Non-misophonic	14.72	13.62	13.17-16.26
Factor 1	Feelings/Isolation	Misophonics	42.71	11.18	40.99-44.43
		Non-misophonic	4.79	6.46	4.06-5.52
Factor 2	Intersocial reactivity	Misophonics	9.37	4.92	8.61-10.13
		Non-misophonic	1.04	1.58	0.86-1.22
Factor 3	Life consequences	Misophonics	10.25	6.09	9.32-11.19
		Non-misophonic	0.94	1.78	0.74-1.14
Factor 4	Avoidance/Repulsion	Misophonics	22.33	4.76	21.60-23.06
		Non-misophonic	7.23	5.56	6.60-7.87
Factor 5	Pain	Misophonics	7.24	5.24	6.44-8.05
		Non-misophonic	0.89	1.86	0.68-1.11

**Table A2.** A Repeat of Table A1 But Now Using Only Our General Population Sample, That is, Means, Standard Deviations (SD) and 95% Confidence Intervals for 45 People From the General Population Identified as Misophonic Using our SMS-Adult, and 284 Identified as Non-Misophonic, in Our Scale and Its Five Factors

Factor	*SMS-Adult*	Misophonic status (from *SMS-Adult*)	*M*	*SD*	Confidence interval
Overall	Total—Part 2	Misophonics	87.38	26.06	85.92-88.83
		Non-misophonic	13.59	12.88	13.37-13.81
Factor 1	Feelings/Isolation	Misophonics	39.78	12.94	38.33-41.23
		Non-misophonic	4.18	5.82	3.96-4.40
Factor 2	Intersocial reactivity	Misophonics	9.64	5.17	8.19-11.09
		Non-misophonic	1.00	1.57	0.78-1.21
Factor 3	Life consequences	Misophonics	10.40	6.04	8.95-11.85
		Non-misophonic	0.87	1.63	0.65-1.08
Factor 4	Avoidance/Repulsion	Misophonics	22.00	4.87	20.55-23.45
		Non-misophonic	6.83	5.42	6.62-7.05
Factor 5	Pain	Misophonics	7.58	4.83	6.13-9.03
		Non-misophonic	0.87	1.86	0.65-1.09

### Part 1

Researchers may wish to present descriptive statistics showing whether participants indicated a trigger within the 48 known trigger items in Part 1. The order of these triggers, from most to least common among our misophonia group, is shown below (but repeated with more detail in Table 5—SI). We found that 98.2% of our misophonics had a trigger within the top 36 ranked triggers, and 99.4% had a trigger within the top 39.

*Misophonia triggers ranked from most to least common*: Chewing, Lip-smacking, Wet mouth sounds, Throat clearing, Slurping, Sniffing, Crunchy foods, Crispy snacks, Swallowing, Foot tapping/on floor, Pen tapping, Pen clicking, Coughing, Some voices, Finger tapping, Snoring, Breathing, Leg rocking, Humming, Whistling, Plastic rustling, Dog barking, Burping, Clock ticking, Paper rustling, Foot shuffling, Typing, Letters, Accents, Consonants, Hiccupping, Sneezing, Snorting, Other eating, Other throat, Car, Other background, Fridge, Other voice, Other nose/mouth, Vowels, Washing machine, Dishwasher, Other tapping, Fan, Other rustling, Chair rocking, Other visual.

## Notes

1. Misophonia has a mismatch between its strict etymological roots (literally a “hatred of sound”) and its slightly broader range of triggers, which include “visual triggers such as . . . jiggling or swinging legs, as well as visual stimuli associated with an auditory trigger, such as watching someone eat” (Swedo et al., 2022, p. 12).

2. It is important to acknowledge that there is a spectrum among people with misophonia, where their experiences can range from debilitating to simply mildly annoying. In this article, we present a measure to separate misophonics from non-misophonics, where the term “misophonia” is understood here to mean clinically significant misophonia. We return to this issue in our discussion.

3. We point out to the reader that these 53 items will be reduced by our factor analysis to leave 39 items in Part 2 of our final measure (see the appendix). These 39 items are also used in the questionnaire for adolescents (SMS-Adolescent; Rinaldi et al., 2022).

4. Four of our 53 items (9, 10, 26, 47; see Table 3) resembled items which were initially considered for, but ultimately did not appear in, the S-Five questionnaire (Vitoratou et al., 2021). There was no overlap between our final scales, and we include these early items with their permission (i.e.,“If I can, I leave the room to avoid telling off people for the sounds they make”; “Some sounds make me so angry that not many things in life can make me feel this way”; “I can feel physical pain if I cannot avoid a sound”; “When I visit friends houses I am afraid that I will have to bear horrible sounds that they and their families make”). Similarly, two of our final items (15 and 38) resembled items from the Misophonia Assessment Questionnaire (“I feel that no one can help me with my sound issues” and “You want to scream or cry loudly”; Johnson, 2014), and we again include these with permission.

5. *N* = 29 from the general population (28 female, 1 male) and *n* = 6 self-referred misophonics (all female) could not be classified due to missing data (making us unable to calculate their SMS-Total score).

6. We remind the reader that the MQ’s Misophonia Severity Scale is a rating from 1 (*minimal*) to 15 (*very severe*) where a threshold score ≥7 indicates clinically signiﬁcant symptoms of misophonia, that is, at least “moderate sound sensitivities” that cause “signiﬁcant interference.”

7. Here we show that misophonics dislike significantly more triggers than controls. But we point out that that non-misophonics also dislike a subset of triggers. This is entirely expected, because misophonia appears to represent an exaggeration of everyday disliking experienced by all people (Simner et al., 2021). It is also possible that our control group may contain sporadic cases of misophonia (see Discussion). As such, our triggers (in Part 1) can only be useful on individual level if they are considered alongside our diagnostic threshold in Part 2. We explain how this might work in our discussion.

8. Similarly, the combined [% sensitivity + % specificity] was 165, when using the MQ for our groupwise division—again meeting the principles for high-value high-quality testing.
