# Supplementary material for: An Automated Online Measure for Misophonia: The Sussex Misophonia Scale for Adults
Source: Assessment. 2024 Feb 27;31(8):1598–614. doi: 10.1177/10731911241234104 (PMC11528938; doi:10.1177/10731911241234104)
Supplement: sj-docx-1-asm-10.1177_10731911241234104 – Supplemental material for An Automated Online Measure for Misophonia: The Sussex Misophonia Scale for Adults [file sj-docx-1-asm-10.1177_10731911241234104.docx]

Supplementary Materials


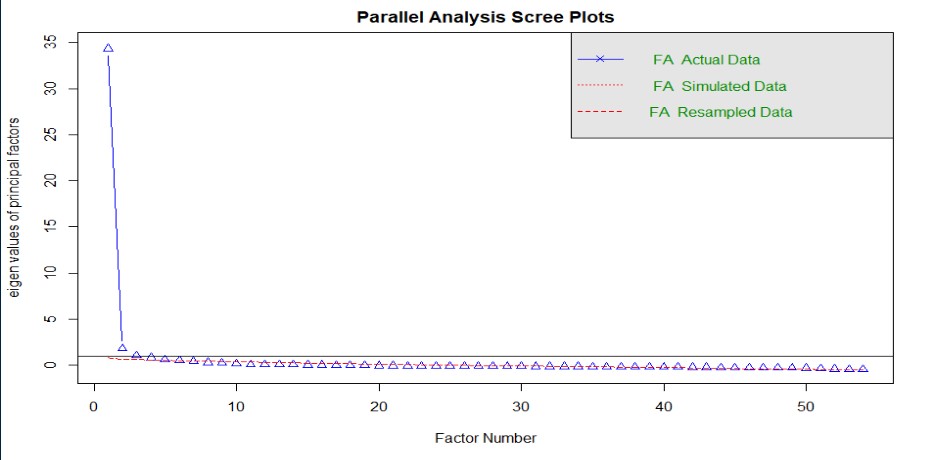


Figure 1. Scree plot showing the parallel analysis

Table 1.

*Summary of existing misophonia questionnaires*

| **Questionnaire** | **Source** | **Validation** | **Items** | **Factor structure** | **Validated threshold** |
| --- | --- | --- | --- | --- | --- |
| Misophonia Assessment Questionnaire (MAQ) | (Dozier, 2015; Johnson, 2014)(Dozier, 2015; Johnson, 2014)(Dozier, 2015; Johnson, 2014) | - | 21 | - | - |
| Misophonia Impact Survey | (Dozier, 2016) | - | 5 | - | - |
| Misophonia Activation Scale (MAS) | (Fitzmaurice, 2010) | - | 10 | - | - |
| Misophonia Physiological Response Scale (MPRS)/ Misophonia Trigger Severity Scale | (Bauman & Dozier, 2013; Dozier, 2015) | - | 12 | - | - |
| Misophonia Coping Responses | (Dozier, 2013a) | - | 21 | - | - |
| Misophonia Emotional Response | (Dozier, 2013b, 2015) | - | 30 | - | - |
| Unnamed misophonia questionnaire | Eric Vernon-Cole, unpublished, online | - | 21 | - | - |
| The Misophonia Questionnaire (MQ) | (Wu et al., 2014) | Yes | 20 | Yes (2) | - |
| Amsterdam Misophonia Scale (A-MISO-S) | (Naylor et al., 2020; Schröder et al., 2013) | Yes | 6 | Yes (1) | - |
| S-Five: a psychometric tool for assessing misophonia | (Vitoratou et al., 2018) unpublished, online | Yes | 85 | -- | - |
| Q**uestionnaires published after development of SMS-Adult** | **Source** | **Validation** | **Items** | **Factor structure** | **Validated threshold** |
| Selective Sound Sensitivity Syndrome Scale (S-Five) | (Vitoratou et al., 2021) | Yes | 62 | Yes (5) | - |
| Duke Misophonia Questionnaire (DMQ) | (Rosenthal et al., 2021) | Yes | 86 | Yes (9) | Yes |
| Misophonia Response Scale | (Dibb et al., 2021) | Yes | 22 | Yes (3) | - |
| MisoQuest | (Siepsiak, Śliwerski, et al., 2020) | Yes | 14 | Yes (1) | - |

^Columns describes the name of the measure, its published status, number of items, whether it has been validated and assessed for factor structure (with number of factors in brackets), and whether its threshold has been validated in any way. All measures above the grey header-bar were available at the time of testing and so their items were incorporated into our questionnaire. Those below the grey bar were conducted simultaneous to our own and appeared in print subsequently.^

Exploratory Factor Analysis

In our manuscript we describe the iterative process of running exploratory factor analyses, and removing items that do not load onto any factor, or that load onto more than one factor above .300. Here we present the factor loadings for the two models preceding our final model presented in the main text.

The first iteration of our exploratory factor analysis is shown in Table 2, with items that were subsequently removed highlighted in grey. The second iteration of exploratory factor analysis is shown in Table 3, again with items subsequently removed highlighted in grey. Our third and final model is presented in the main manuscript.

Table 2. *Five-factor Model Loadings.* Table shows first iteration of our factor analysis with all items in Part 2 of our measure (Likert-scale items) and their factor loadings. Bold represents factor loadings >3.00, grey cells show items that were removed due to cross-loading onto multiple factors or not loading onto any factor. Items are sorted by highest loading.

| No. | Item | 1 | 2 | 3 | 4 | 5 |
| --- | --- | --- | --- | --- | --- | --- |
| 23 | I feel embarrassed about hating certain sounds | **0.84** | -0.01 | 0.07 | -0.09 | 0.1 |
| 15 | I worry nobody can help with my sound problems | **0.81** | 0.04 | 0.05 | 0.13 | 0 |
| 30 | I'm worried about always having problems from hearing certain sounds | **0.72** | 0.19 | 0.04 | 0.07 | 0.01 |
| 25 | Hatred of some sounds make me feel lonely | **0.63** | 0.21 | 0.13 | 0.08 | -0.07 |
| 13 | I feel guilty because of my reaction to sounds | **0.61** | 0 | 0.16 | -0.11 | 0.29 |
| 4 | I feel no one really understands that I have a problem with sounds | **0.61** | 0 | 0.09 | 0.1 | 0.23 |
| 42 | I suspect my friends think I'm weird, because of my reaction to sounds | **0.6** | 0.16 | 0.06 | 0.14 | -0.02 |
| 35 | People think I'm crazy because of my reaction to sounds | **0.58** | 0.01 | 0.1 | 0.27 | 0.06 |
| 24 | Nobody believes my problem with sounds | **0.57** | -0.05 | 0.14 | 0.27 | 0.04 |
| 21 | My life is worse than friends who don't find sounds difficult | **0.53** | **0.32** | 0.12 | 0.11 | -0.09 |
| 33 | My life is worse because of sound problems | **0.53** | 0.30 | 0.14 | 0.13 | -0.06 |
| 31 | I try not to let people know I hate certain sounds | **0.52** | -0.01 | 0.07 | -0.02 | **0.3** |
| 8 | Problems with sounds affect my work life, home life or social life | **0.51** | **0.34** | -0.01 | 0.07 | 0.14 |
| 51 | I think my problems with  sounds are getting worse with age | **0.49** | 0.18 | 0.05 | 0.21 | 0.06 |
| 20 | I know my reaction to  sounds is extreme | **0.49** | 0.08 | 0.08 | 0.05 | **0.34** |
| 6 | Other people make fun of me for hating sounds | **0.47** | -0.09 | 0.15 | 0.2 | 0.15 |
| 3 | I have a problem because hearing certain sounds makes me unhappy | **0.45** | 0.14 | 0.06 | 0.13 | 0.24 |
| 10 | Not many things in life make me as angry as some sounds | **0.44** | 0.05 | -0.03 | **0.32** | 0.26 |
| 14 | Some sounds make me want to run away from them | **0.38** | 0.12 | 0.15 | 0.04 | **0.39** |
| 38 | Some sounds make me want to scream or cry | **0.38** | 0.07 | 0.19 | 0.16 | 0.27 |
| 16 | Hatred of some sounds makes me want to avoid people | **0.37** | 0.18 | 0.02 | 0.24 | 0.27 |
| 40 | The sounds I don’t like make me feel afraid | **0.35** | 0.23 | **0.33** | -0.07 | -0.08 |
| 5 | I feel scared hearing sounds I don’t like | **0.35** | 0.2 | 0.29 | -0.06 | 0.02 |
| 47 | When I visit friends' houses, I'm scared there will be horrible sounds | **0.31** | **0.42** | 0.15 | 0.16 | -0.07 |
| 1 | Sounds that other people don't mind can make me really angry | 0.26 | 0.08 | 0.02 | 0.22 | **0.44** |
| 52 | Certain sounds make me feel rage | 0.26 | 0.01 | 0.05 | **0.45** | **0.31** |
| 34 | Hearing certain sounds makes me unable to control feelings of anger | 0.22 | 0.1 | 0.01 | **0.41** | **0.32** |
| 28 | Problems with sounds has meant I don't have many friends | 0.21 | **0.57** | 0.12 | 0.14 | -0.15 |
| 9 | Sometimes I leave the room, to avoid telling people off for making bad sounds | 0.21 | 0.13 | 0.09 | 0.12 | **0.5** |
| 44 | I don't get on well with some family members because of the sounds these family members make | 0.2 | 0.16 | 0.17 | **0.31** | 0.17 |
| 49 | I feel like people make sounds  on purpose just to upset me | 0.2 | -0.08 | 0.2 | **0.56** | 0.02 |
| 53 | I put on headphones to block out certain sounds | 0.18 | 0.18 | 0.01 | 0.03 | **0.48** |
| 7 | It hurts when I hear certain sounds, even if it doesn't hurt other people | 0.16 | 0.1 | **0.69** | -0.08 | 0.08 |
| 43 | I react more strongly to some sounds if I'm having a bad day | 0.16 | 0.1 | -0.01 | 0.07 | **0.54** |
| 46 | I say things aloud in order to avoid listening to bad sounds | 0.14 | 0.13 | 0.13 | 0.09 | **0.38** |
| 39 | I hate some sounds so much, I want to scream at people who make them | 0.13 | 0.08 | 0.1 | **0.43** | **0.35** |
| 27 | The sound made by some people makes me feel the need to avoid them | 0.12 | 0.22 | 0.03 | 0.30 | **0.40** |
| 18 | I try to avoid going to people's houses if those people make sounds I hate | 0.1 | **0.42** | 0.10 | 0.22 | 0.17 |
| 50 | I want to get pay back on people who make certain sounds | 0.08 | 0.07 | 0.02 | **0.76** | -0.09 |
| 22 | I hate people who make sounds I don't like | 0.07 | 0.22 | 0.01 | **0.62** | 0.08 |
| 29 | My hatred of some sounds creates problems in work | 0.04 | **0.83** | 0.08 | 0.01 | -0.01 |
| 26 | I feel physical pain if unable to avoid a sound | 0.04 | 0 | **0.94** | 0.01 | -0.03 |
| 45 | I copy people to show them how annoying their sounds are | 0.02 | 0.06 | 0.12 | 0.23 | 0.22 |
| 41 | I don't like work because there are lots of sounds I hate | 0.01 | **0.88** | -0.02 | 0.01 | 0.06 |
| 2 | Certain sounds make me feel disgusted, even if those sounds don't disgust other people | 0.01 | 0.13 | 0.15 | 0.12 | **0.53** |
| 36 | I cover my ears to block out certain sounds | 0 | 0.08 | 0.23 | -0.01 | **0.53** |
| 37 | I've told some people they must not make certain noises around me | -0.02 | 0.12 | 0.18 | 0.25 | **0.39** |
| 19 | I try to avoid going to work because of difficulties with sounds | -0.03 | **0.8** | 0.08 | 0.08 | -0.04 |
| 32 | I feel pain on my body when I hear certain sounds | -0.05 | -0.04 | **1** | 0.01 | -0.01 |
| 17 | I don't do well at work because of distractions from sounds | -0.06 | **0.73** | 0.06 | -0.01 | 0.21 |
| 11 | There are some sounds I hate so much, I shout at people | -0.06 | 0.06 | 0.16 | **0.49** | 0.21 |
| 12 | Sounds often cause me physical pain | -0.07 | 0.04 | **0.93** | 0.03 | 0 |
| 48 | I want to hurt people who make sounds I hate | -0.08 | 0.11 | 0.13 | **0.76** | 0.01 |

Table 3. *Five-factor Model Loadings.* Table shows first iteration of our factor analysis with all items in Part 2 of our measure (Likert-scale items) and their factor loadings. Bold represents factor loadings >3.00, grey cells show items that were removed due to cross-loading onto multiple factors or not loading onto any factor. Items are sorted by highest loading.

| No. | Item | 1 | 2 | 3 | 4 | 5 |
| --- | --- | --- | --- | --- | --- | --- |
| 23 | I feel embarrassed about hating certain sounds | **0.92** | 0.04 | -0.01 | 0.05 | -0.12 |
| 15 | I worry nobody can help with my sound problems | **0.88** | 0.01 | 0.03 | -0.04 | 0.1 |
| 30 | I'm worried about always having problems from hearing certain sounds | **0.77** | 0.02 | 0.19 | -0.03 | 0.06 |
| 13 | I feel guilty because of my reaction to sounds | **0.7** | 0.12 | 0.01 | 0.21 | -0.14 |
| 25 | Hatred of some sounds make me feel lonely | **0.68** | 0.11 | 0.2 | -0.1 | 0.07 |
| 4 | I feel no one really understands that I have a problem with sounds | **0.66** | 0.06 | 0 | 0.21 | 0.07 |
| 35 | People think I'm crazy because of my reaction to sounds | **0.64** | 0.07 | 0.02 | 0.04 | 0.23 |
| 42 | I suspect my friends think I'm weird, because of my reaction to sounds | **0.63** | 0.04 | 0.15 | -0.02 | 0.14 |
| 24 | Nobody believes my problem with sounds | **0.6** | 0.11 | -0.05 | 0.06 | 0.25 |
| 31 | I try not to let people know I hate certain sounds | **0.58** | 0.03 | -0.02 | 0.26 | -0.04 |
| 33 | My life is worse because of sound problems | **0.53** | 0.14 | 0.28 | -0.04 | 0.12 |
| 51 | I think my problems with  sounds are getting worse with age | **0.52** | 0.05 | 0.17 | 0.08 | 0.17 |
| 3 | I have a problem because hearing certain sounds makes me unhappy | **0.5** | 0.04 | 0.13 | 0.24 | 0.09 |
| 6 | Other people make fun of me for hating sounds | **0.49** | 0.13 | -0.11 | 0.18 | 0.2 |
| 38 | Some sounds make me want to scream or cry | **0.46** | 0.16 | 0.06 | 0.23 | 0.13 |
| 16 | Hatred of some sounds makes me want to avoid people | **0.39** | 0.01 | 0.16 | **0.32** | 0.2 |
| 5 | I feel scared hearing sounds I don’t like | **0.35** | 0.26 | 0.14 | 0 | 0.02 |
| 1 | Sounds that other people don't mind can make me really angry | **0.34** | -0.01 | 0.09 | **0.42** | 0.14 |
| 9 | Sometimes I leave the room, to avoid telling people off for making bad sounds | 0.24 | 0.07 | 0.11 | **0.52** | 0.09 |
| 43 | I react more strongly to some sounds if I'm having a bad day | 0.22 | -0.04 | 0.08 | **0.54** | 0.03 |
| 44 | I don't get on well with some family members because of the sounds these family members make | 0.21 | 0.16 | 0.15 | 0.25 | 0.25 |
| 53 | I put on headphones to block out certain sounds | 0.21 | -0.01 | 0.15 | **0.47** | 0.03 |
| 49 | I feel like people make sounds  on purpose just to upset me | 0.2 | 0.17 | -0.12 | 0.1 | **0.59** |
| 46 | I say things aloud in order to avoid listening to bad sounds | 0.2 | 0.11 | 0.09 | **0.39** | 0.06 |
| 7 | It hurts when I hear certain sounds, even if it doesn't hurt other people | 0.18 | **0.68** | 0.08 | 0.06 | -0.04 |
| 28 | Problems with sounds has meant I don't have many friends | 0.18 | 0.13 | **0.53** | -0.09 | 0.16 |
| 27 | The sound made by some people makes me feel the need to avoid them | 0.13 | 0.02 | 0.2 | **0.49** | 0.25 |
| 18 | I try to avoid going to people's houses if those people make sounds I hate | 0.09 | 0.1 | **0.39** | 0.25 | 0.2 |
| 29 | My hatred of some sounds creates problems in work | 0.07 | 0.09 | **0.84** | -0.04 | -0.01 |
| 22 | I hate people who make sounds I don't like | 0.07 | 0 | 0.2 | 0.15 | **0.6** |
| 50 | I want to get pay back on people who make certain sounds | 0.07 | -0.01 | 0.04 | -0.06 | **0.82** |
| 26 | I feel physical pain if unable to avoid a sound | 0.06 | **0.93** | 0 | -0.03 | 0 |
| 41 | I don't like work because there are lots of sounds I hate | 0.03 | -0.03 | **0.9** | 0.05 | -0.01 |
| 36 | I cover my ears to block out certain sounds | 0.02 | 0.22 | 0.03 | **0.53** | 0.02 |
| 2 | Certain sounds make me feel disgusted, even if those sounds don't disgust other people | 0.01 | 0.13 | 0.1 | **0.61** | 0.08 |
| 37 | I've told some people they must not make certain noises around me | 0 | 0.17 | 0.09 | **0.44** | 0.22 |
| 11 | There are some sounds I hate so much, I shout at people | -0.02 | 0.16 | 0.06 | 0.26 | **0.4** |
| 17 | I don't do well at work because of distractions from sounds | -0.02 | 0.05 | **0.72** | 0.18 | -0.01 |
| 32 | I feel pain on my body when I hear certain sounds | -0.03 | **0.99** | -0.03 | -0.01 | 0 |
| 19 | I try to avoid going to work because of difficulties with sounds | -0.03 | 0.09 | **0.78** | -0.03 | 0.09 |
| 48 | I want to hurt people who make sounds I hate | -0.05 | 0.12 | 0.12 | 0.08 | **0.7** |
| 12 | Sounds often cause me physical pain | -0.06 | **0.93** | 0.05 | 0 | 0.01 |

Table 4. Showing factor correlations for our sample included in our final factor analysis presented in the paper.

|  | Feelings/ Isolation | Life consequences | Intersocial reactivity | Avoidance/ Repulsion | Pain |
| --- | --- | --- | --- | --- | --- |
| Feelings/ Isolation | 1 | .65 | .67 | .70 | .66 |
| Life consequences | .65 | 1 | .42 | .44 | .46 |
| Intersocial reactivity | .67 | .42 | 1 | .68 | .57 |
| Avoidance/ Repulsion | .70 | .44 | .68 | 1 | .64 |
| Pain | .66 | .46 | .57 | .64 | 1 |

Table 5.

*Ranking of Triggers, and the Number and Percent of Misophonics Captured at each Step of Ranking.* Table shows misophonia triggers (hated sounds) ranked from highest (most common among SMS-Misophonics) to lowest. Also shown are the cumulative number (n) and percentage (%) of misophonics captured by each successive step in the ranking, when misophonics are represented by one trigger each (their highest in the ranking). For example, the table shows that 99.4% of misophonics (i.e., 164 out of 165) have at least one disliked sound within the top 38.5 ranked triggers. Trigger names are abbreviated where necessary but full names are shown in the main manuscript. Gaps in the table represent triggers previously assumed by researchers but in fact finding no agreement from the 165 misophonics tested in our study.

|  |  | Cumulative | |  |  | Cumulative | |
| --- | --- | --- | --- | --- | --- | --- | --- |
| Trigger Ranking | Trigger | n | % | Trigger Ranking | Trigger | n | % |
| 1.5 | Chewing | 131 | 79.4 | 26 | Foot shuffling |  |  |
| 1.5 | Lip smacking | 140 | 84.8 | 27 | Typing |  |  |
| 3 | Wet mouth sounds |  |  | 28 | Letters |  |  |
| 4 | Throat clearing | 149 | 90.3 | 29 | Accents |  |  |
| 5 | Slurping | 150 | 90.9 | 30.5 | Consonants |  |  |
| 6 | Sniffing |  |  | 30.5 | Hiccupping |  |  |
| 7 | Crunchy foods | 151 | 91.5 | 32 | Sneezing |  |  |
| 8 | Crispy snacks |  |  | 33 | Snorting |  |  |
| 9 | Swallowing |  |  | 34 | Other [eating] |  |  |
| 10 | Foot tapping / on floor | 156 | 94.5 | 35 | Other [throat] |  |  |
| 11 | Pen tapping |  |  | 36.5 | Car | 162 | 98.2 |
| 12 | Pen clicking |  |  | 36.5 | Other [back-ground] |  |  |
| 13 | Coughing |  |  | 38.5 | Fridge |  |  |
| 14 | Some voices | 160 | 97.0 | 38.5 | Other [voice] | 164 | 99.4 |
| 15 | Finger tapping |  |  | 40 | Other [nose] |  |  |
| 16 | Snoring |  |  | 41 | Vowels |  |  |
| 17 | Breathing |  |  | 42 | Washing machine |  |  |
| 18 | Leg rocking |  |  | 43 | Dishwasher |  |  |
| 19 | Humming |  |  | 44 | Other [tapping |  |  |
| 20 | Whistling |  |  | 45 | Fan |  |  |
| 21 | Plastic rustling | 161 | 97.6 | 46 | Other_rustling |  |  |
| 22 | Dog barking |  |  | 47.5 | Chair rocking |  |  |
| 23 | Burping |  |  | 47.5 | Other [visual] |  |  |
| 24.5 | Clock ticking |  |  |  |  |  |  |
| 24.5 | Paper rustling |  |  |  | 1 person had no listed triggers | | |

Table 6.

*Correlation Matrix showing reliability between scores on the Sussex Misophonia Scale and the MQ. This table is similar to Table 3 in the main manuscript but here we combine both groups (misophonics and controls) into a single population.*

|  | | SMS Total | SMS (non-trigger) Factors | | | | | MQ total | MQ Sub-scales | | |
| --- | --- | --- | --- | --- | --- | --- | --- | --- | --- | --- | --- |
|  |  |  | **1** | **2** | **3** | **4** | 5 |  | Triggers | Emotion behavior | Severity |
| SMS Total | | 1 | 0.98 | 0.88 | 0.80 | 0.87 | 0.92 | 0.85 | 0.59 | 0.90 | 0.78 |
| SMS (non-trigger) Factors | **1** | 0.98 | 1 | 0.82 | 0.75 | 0.80 | 0.87 | 0.82 | 0.54 | 0.87 | 0.75 |
|  | **2** | 0.88 | 0.82 | 1 | 0.72 | 0.77 | 0.76 | 0.75 | 0.55 | 0.79 | 0.72 |
|  | **3** | 0.80 | 0.75 | 0.72 | 1 | 0.64 | 0.65 | 0.62 | 0.49 | 0.64 | 0.61 |
|  | **4** | 0.87 | 0.80 | 0.77 | 0.64 | 1 | 0.77 | 0.78 | 0.45 | 0.84 | 0.69 |
|  | **5** | 0.92 | 0.87 | 0.76 | 0.65 | 0.77 | 1 | 0.85 | 0.65 | 0.88 | 0.72 |
| MQ total | | 0.85 | 0.85 | 0.82 | 0.75 | 0.62 | 0.78 | 0.85 | 1 | 0.87 | 0.96 |
| MQ Sub-scales | Triggers | 0.59 | 0.54 | 0.55 | 0.49 | 0.45 | 0.65 | 0.87 | 1 | 0.69 | 0.65 |
|  | Emotion behavior | 0.90 | 0.87 | 0.79 | 0.64 | 0.84 | 0.88 | 0.96 | 0.69 | 1 | 0.78 |
|  | Severity | 0.78 | 0.75 | 0.72 | 0.61 | 0.69 | 0.72 | 0.81 | 0.65 | 0.78 | 1 |

^Table shows total scores on each sub-scale, as well as the SMS factors (1=Feelings/ Isolation; 2=Life consequences; 3=Intersocial reactivity; 4=Avoidance/ Repulsion; 5=Pain) and MQ Sub-scales. Note that the MQ Sub-scale here called “triggers” is named ‘symptoms’ at source (Wu et al., 2014) and that no SMS factors relate to triggers. This explains the lower correlations between SMS factors and the MQ triggers sub-scale. All other correlations are high, both within our own sub-scales (dark grey shading) and between measures (no shading). Light grey shadings show our data for correlations within the MQ.^ *^Note.^* ^All correlations are significant at^ *^p^* ^<.001 except where otherwise shown.^
